# A Novel Affinity Tag, ABTAG, and Its Application to the Affinity Screening of Single-Domain Antibodies Selected by Phage Display

**DOI:** 10.3389/fimmu.2017.01406

**Published:** 2017-10-30

**Authors:** Greg Hussack, Toya Nath Baral, Jason Baardsnes, Henk van Faassen, Shalini Raphael, Kevin A. Henry, Jianbing Zhang, C. Roger MacKenzie

**Affiliations:** ^1^Human Health Therapeutics Research Centre, National Research Council Canada, Ottawa, ON, Canada; ^2^Human Health Therapeutics Research Centre, National Research Council Canada, Montréal, QC, Canada

**Keywords:** antibody discovery, phage display, surface plasmon resonance, single-domain antibody, nanobody, V_H_H, carcinoembryonic antigen-related cell adhesion molecule 6

## Abstract

ABTAG is a camelid single-domain antibody (sdAb) that binds to bovine serum albumin (BSA) with low picomolar affinity. In surface plasmon resonance (SPR) analyses using BSA surfaces, bound ABTAG can be completely dissociated from the BSA surfaces at low pH, over multiple cycles, without any reduction in the capacity of the BSA surfaces to bind ABTAG. A moderate throughput, SPR-based, antibody screening assay exploiting the unique features of ABTAG is described. Anti-carcinoembryonic antigen-related cell adhesion molecule 6 (CEACAM6) sdAbs were isolated from a phage-displayed sdAb library derived from the heavy chain antibody repertoire of a llama immunized with CEACAM6. Following one or two rounds of panning, enriched clones were expressed as ABTAG fusions in microtiter plate cultures. The sdAb-ABTAG fusions from culture supernatants were captured on BSA surfaces and CEACAM6 antigen was then bound to the captured molecules. The SPR screening method gives a read-out of relative expression levels of the fusion proteins and kinetic and affinity constants for CEACAM6 binding by the captured molecules. The library was also panned and screened by conventional methods and positive clones were subcloned and expressed for SPR analysis. Compared to conventional panning and screening, the SPR-based ABTAG method yielded a considerably higher diversity of binders, some with affinities that were three orders of magnitude higher affinity than those identified by conventional panning.

## Introduction

Over the past 25 years, *in vitro* display technologies, most notably phage and yeast display, have increasingly become the methods of choice for the isolation and affinity maturation of monoclonal antibody fragments. This is especially the case in instances where antibodies with particular properties are required (1, 2), as *in vitro* display technologies allow for the selection process to be biased toward desired outcomes (3). To take full advantage of the power of these technologies it is preferable to screen relatively large numbers of clones after one or two selection cycles to increase the odds of identifying clones with the desired properties. There is ongoing interest, therefore, in the development of more effective and rapid methods for screening antibodies for expression level and for target antigen specificity and affinity.

The classical protocol for isolating antibody fragments against a given target by phage display technology generally includes immunizing an animal, measuring the immune response, constructing a phage-displayed antibody fragment library and performing three to five rounds of panning to enrich for clones that bind to the target. This generally gives a manageable number of clones for subsequent expression, purification and characterization. The antibody fragments can be fragments antigen-binding (Fabs) (4), single-chain variable fragments (scFvs) (4), or single-domain antibodies (sdAbs). sdAbs can be the variable domains (V_H_Hs) of camelid heavy chain antibodies (5, 6), the variable domains (V_NAR_s) of shark immunoglobulin new antigen receptors (7), the variable heavy domains (V_H_s) of conventional antibodies (8, 9), or the variable light domains (V_L_s) of conventional antibodies (10). After panning, phage ELISA is performed on randomly picked clones to identify the leads. Leads are typically expressed and purified for affinity measurements and functional characterization. This is a relatively slow, costly and laborious process that generally yields no more than a dozen binders, and often fewer.

**Figure 1:**
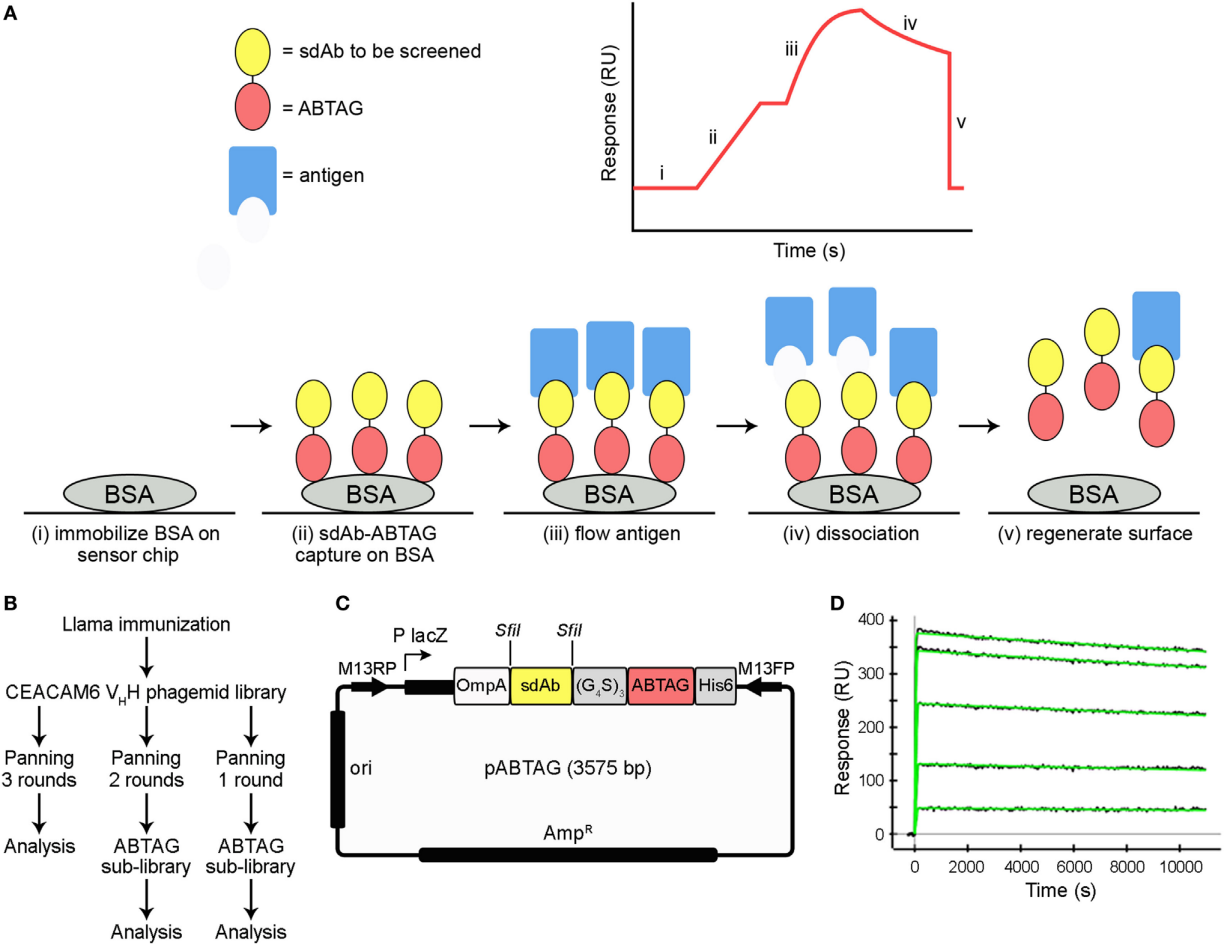
Summary of ABTAG technology and single-domain antibody (sdAb) isolation. Schematic diagram of the ABTAG/surface plasmon resonance (SPR)-based screening method for the evaluation of enriched sdAb clones from phage display panning **(A)**. While anti-CEACAM6 sdAb clones obtained after three rounds of panning were screened by phage ELISA, pools of phage clones enriched after either one or two rounds of phage display library panning were directly subcloned into pABTAG (3,575 bp, representative sdAb of average length), creating two sublibraries, for expression as sdAb-ABTAG fusions by capture on BSA surfaces **(B,C)**. A representative sensorgram demonstrates sdAb-ABTAG fusion proteins are stably retained by BSA surfaces because of an extremely slow dissociation rate **(D)**. BSA, bovine serum albumin; RU, resonance unit.

In this study, we compare different approaches to the isolation sdAbs against carcinoembryonic antigen-related cell adhesion molecule 6 (CEACAM6) from a phage-displayed immune llama V_H_H library. We have used a classical panning protocol followed by subcloning, expression and purification of sdAbs for surface plasmon resonance (SPR) analyses. We also describe a screening method in which anti-CEACAM6 sdAbs are fused to an anti-bovine serum albumin (anti-BSA) sdAb characterized by extremely tight, but readily reversible, binding to BSA. The sdAb, initially termed BSA12, has a *K*_D_ of 4 pM and a *k*_d_ of 9 × 10^−6^ s^−1^, equivalent to an sdAb:BSA half-life of approximately 21 h, yet it can be completely dissociated from BSA surfaces with a 3 s pulse of 100 mM HCl (11, 12). In the SPR-based screening method described here (Figure 1A), panning-enriched sdAb clones are expressed in fusion with the sdAb BSA12 and captured on BSA surfaces for CEACAM6 binding measurements. For applications such as this, we have given the anti-BSA sdAb BSA12 a new designation, ABTAG, with “AB” alluding to the fact that it is an antibody fragment or, alternatively, that it is an albumin-binding “TAG.” With this new methodology, we isolated several extremely high-affinity anti-CEACAM6 sdAbs that were missed by classical panning and screening of the same phage-displayed library. The ABTAG technology should have general utility in the isolation of antibody fragments from phage display libraries—i.e., it could be applied to V_L_, V_H_, V_NAR_, scFv, and Fab libraries. ABTAG technology has previously been used to screen sdAbs specific to human Fc (fragment crystallizable) region (13).

## Materials and Methods

### Immunization and Serology

A llama (*Lama glama*) was immunized five times (days 1, 21, 35, 49, and 63) subcutaneously with approximately 100 µg of recombinant CEACAM6 [N- and A-domains, residues 35–232; (14)] per injection, kindly provided by Helix BioPharma (Aurora, ON, Canada), with Complete Freund’s Adjuvant on day 1 and Incomplete Freund’s Adjuvant on the remaining days. On days 1, 22, 36, 49, 64, and 71 blood (50 mL) was collected, from which sera and peripheral blood lymphocytes were isolated (15). This study was carried out in accordance with Animal Use Protocols approved by the National Research Council Canada Animal Care Committee.

Microtiter plates were coated with 10 µg/mL of CEACAM6 overnight at 4°C in 15 mM Na_2_CO_3_, pH 9.6, followed by blocking with 2% fat-free dry milk (BioRad Laboratories, Mississauga, ON, Canada) in PBS. Serially diluted serum was then added to the wells. Detection of llama IgGs was performed with goat anti-llama IgG (Bethyl Laboratories, Montgomery, TX, USA) and a horseradish peroxidase anti-goat IgG conjugate (Cedarlane Laboratories, Burlington, ON, Canada). Finally, peroxidase substrate (KPL, Gaithersburg, MD) was added followed by 1 M H_3_PO_4_ after 15 min. The absorbance at 450 nm was then measured.

### Phage-Displayed Library Construction

RNA was extracted from peripheral blood lymphocytes obtained from blood drawn on day 71 by using a QIAamp RNA Blood Mini Kit (Qiagen Inc., Mississauga, ON, Canada). cDNA was synthesized by using a first-strand cDNA synthesis kit (GE Healthcare, Mississauga, ON, Canada). Sense primers MJ1, MJ2, and MJ3 and antisense primers CH2 and CH2b3 (15) were used to amplify V_H_H and V_H_-C_H_1 encoding regions (600 and 900 bp, respectively). These two fragments were separated by agarose gel electrophoresis and the V_H_H band was purified from the gel. Nested PCR, using primers MJ7 and MJ8 (15), was performed to amplify all V_H_Hs. The final PCR fragments were ligated into the phagemid vector pMED1 (16) using *Sfi*I restriction sites. The ligated vector was used to transform electrocompetent *Escherichia coli* TG1 cells.

### Selection of sdAbs by Conventional Panning

The V_H_H repertoire was expressed on phage surfaces after rescuing with M13K07 helper phage. Three rounds of phage display panning were conducted (Figure 1B) as described (17). Briefly, specific V_H_Hs against CEACAM6 were enriched by *in vitro* selection on microtiter plates coated with antigen (10 µg/mL). Phage particles eluted with 100 mM triethylamine, pH 11, were immediately neutralized with 1 M Tris–HCl, pH 7.4, and used to infect exponentially growing TG1 cells. To assess the enrichment of phage particles carrying antigen-specific V_H_Hs, serial dilutions of the phage eluted from antigen coated *vs* non-coated control wells were used to infect exponentially growing TG1 cells. Individual colonies randomly picked after all three rounds of panning were tested for binding to CEACAM6 by phage ELISA according to standard procedures (17). Unique clones identified by DNA sequencing were subcloned in pSJF2H, expressed, and purified, as described previously (15), for SPR analysis.

### Subcloning Phage-Displayed sdAbs for sdAb-ABTAG Expression

Phage ELISA was performed with 20–50 monoclonal phage eluted from rounds 1 and 2 to estimate the percentage of phage clones binding to antigen. Eluted phage pools were then used for the construction of two sublibraries, one from round 1 eluted phage and a second from round 2 eluted phage, in which enriched clones were fused to ABTAG in a vector designed for this purpose (Figures 1B,C). Briefly, DNA encoding the V_H_Hs was amplified from eluted phage pools with the introduction of *Sfi*I restriction sites at both ends of the fragments. The PCR fragments were then ligated into the ABTAG fusion vector. Individual clones were inoculated into LB medium supplemented with 100 µg/mL ampicillin in 96-well microtiter plates and grown at 37°C overnight with shaking. Supernatants were collected after centrifugation of the cell cultures for ELISA and SPR.

### SPR Analysis of sdAb-ABTAG Binding Affinity to BSA

For ProteOn (BioRad Laboratories Canada Inc., Mississauga, ON, Canada) analysis of sdAb-ABTAG affinity for BSA, covalently immobilized BSA surfaces were prepared using NHS/*N*-ethyl-*N*′-(3-diethylaminopropyl) carbodiimide hydrochloride (EDC) coupling. Immobilization reagents, *N*-sulfohydroxysuccinimide (sNHS), EDC, and ethanolamine were from the ProteOn Amine Coupling Kit (BioRad Laboratories Canada Inc.). The BSA capture surface was created by immobilizing 6.0 µg/mL Fraction V BSA (EMD Chemicals Inc., Gibbstown, NJ, USA) diluted in 10 mM sodium acetate buffer, pH 4.5, onto a GLM chip activated by a 1:10 dilution of sNHS/EDC that was injected for 300 s at 30 µL/min in the vertical direction as recommended by the manufacturer. Approximately 1,700 resonance units (RUs) of a 2.5 µg/mL BSA solution in 10 mM sodium acetate, pH 4.5, were then immobilized in the vertical direction in order to generate blank control spots in the horizontal direction for referencing. Surfaces were quenched with a 300 s injection of 1 M ethanolamine at 30 µL/min. For affinity determination, a 200 nM threefold dilution series of an sdAb-ABTAG fusion protein clone with buffer blank was injected in triplicate over the BSA surface. Each injection was carried out at 50 µL/min for 120 s with a 3 h dissociation. All experiments were carried out at 25°C and in running buffer consisting of 10 mM HEPES, pH 7.4, containing 150 mM NaCl, 0.05% Tween 20, and 3 mM EDTA. Data from the three independent sensorgrams were double referenced to the control spots and independently fitted to a 1:1 interaction model after determining that the binding was not mass-transport limited.

### Screening of CEACAM6 Binding to sdAb-ABTAG Fusions by Dissociation Rate Constants

The binding of CEACAM6 to sdAb-ABTAG fusions captured on immobilized BSA was determined by SPR using a Biacore 3000 (GE Healthcare). Approximately 8,000 RUs of BSA (Sigma-Aldrich Canada, Oakville, ON, Canada) were immobilized on all four flow cells (Fcs) of CM5 sensor chip (GE Healthcare). Immobilizations were carried out at a protein concentration of 50 µg/mL in 10 mM acetate buffer, pH 4.5, using an amine coupling kit (GE Healthcare). Forty microliters of the *E. coli* culture supernatants of 48 randomly picked clones from the round 2 sublibrary, containing the anti-CEACAM6 sdAb-ABTAG fusions, were added to a 96-well microtiter plate and covered with self-adhesive foil (GE Healthcare). Sixty microliters of running buffer (10 mM HEPES, pH 7.4, containing 150 mM NaCl, 3 mM EDTA, and 0.01% surfactant P20) were added to each well to dilute the culture supernatants, followed by mixing. As the Biacore 3000 has four Fcs, three fusions were simultaneously analyzed to increase screening throughput—the fourth Fc served as an ABTAG reference surface. ABTAG and the fusions were captured on the BSA surfaces by sequentially injecting 40 µL of three different diluted culture supernatants over Fcs 2, 3, and 4 at a flow rate of 5 µL/min. For some clones over 4,000 RUs of the sdAb-ABTAG fusions were captured in this manner. For the reference surface, 20 µL of 80 nM ABTAG were injected over Fc 1, followed by injection of a 60 µL buffer blank. Next, 1 µM of CEACAM6 was injected over all four Fcs at a flow rate of 20 µL/min and the dissociations were monitored for 3 min followed by surface regeneration with a 15 s injection of 10 mM glycine/HCl, pH 2.0. In all instances, analyses were carried out at 25°C in running buffer. The reference subtracted data were aligned and a corresponding buffer blank was subtracted from each sensorgram. The dissociation phase was normalized with the highest response in each sensorgram set at 100% and no response set at 0% for all data sets to rank binders by their dissociation rate. This qualitative analysis allowed for easy visual identification of binders with fast, medium and slow dissociation rates, which generally correlate with low, medium, and high affinities. Off-rates were determined using the BIAevaluation v4.1.1 Software (GE Healthcare). DNA sequencing of CEACAM6 binding sdAb-ABTAG clones was then performed as described previously (15).

### Screening of CEACAM6 Binding to sdAb-ABTAG Fusions by Full Kinetic Analysis

Full kinetic data for CEACAM6 binding to captured sdAb-ABTAG fusions were obtained by single-cycle kinetics (SCK) using a Biacore T200 (GE Healthcare). The same 48 clones (round 2 sublibrary) that were screened by off-rate analysis above were analyzed in this manner. Fresh microtiter plate cultures were grown as described above. Cultures were harvested and periplasmic extracts prepared as described (15). Approximately 9,500 RUs of BSA (Sigma-Aldrich Canada) were immobilized on all four Fcs of CM5 Series S sensor chips (GE Healthcare). Immobilizations were carried out at a protein concentration of 50 µg/mL in 10 mM acetate buffer, pH 4.5, using an amine coupling kit (GE Healthcare). Fifty microliter of the periplasmic extracts of the 48 clones and 100 µL of running buffer (10 mM HEPES, pH 7.4, 150 mM NaCl, 3 mM EDTA, 0.05% surfactant P20) were added to a 96-well microtiter plate. ABTAG (80 nM) was captured on Fc 1 at a flow rate of 5 µL/min for 4 min, as a reference, and the diluted periplasmic extracts of three clones were captured on Fcs 2, 3, and 4 at a flow rate of 5 µL/min for 8 min per cycle. CEACAM6 was injected over all four Fcs at concentrations of 0.1, 1, 10, 100, and 1,000 nM at a flow rate of 5 µL/min for 3 min at 25°C in running buffer. Dissociations were monitored for 5 min followed by surface regeneration with 10 mM glycine/HCl, pH 2.0, at a flow rate of 10 µL/min for 60 s. Buffer-blank cycles preceded each antigen injection cycle. Data were analyzed using Biacore T200 Evaluation Software v3.0 (GE Healthcare).

Moderate-throughput sdAb screening with affinity and kinetic determination was also carried out using the BioRad ProteOn SPR instrument (BioRad Laboratories Canada Inc.). Microtiter plate cultures of 576 randomly picked clones from the round 1 sublibrary were centrifuged and the supernatants analyzed for binding to CEACAM6 by ELISA. ELISA-positive clones were sequenced (15) and subjected to ProteOn screening. Approximately 1,200 RUs of BSA were immobilized, as described above, except for immobilizing the BSA in the horizontal direction to create horizontal referencing spots with a BSA surface. The screening procedure occurred in two steps with a ligand capture in the vertical direction followed by an analyte injection in the horizontal direction. One buffer injection for 30 s at flow 100 µL/min in the ligand direction was used to stabilize the baseline after switching from the previous analyte injection. For each ligand capture, five individual *E. coli* culture supernatants each containing an sdAb-ABTAG fusion were diluted 1:25 in running buffer containing 1 mg/mL carboxymethyldextran sodium salt (Sigma-Aldrich Canada) to reduce non-specific protein interactions with the GLM chip surface and injected for 240 s at a flow rate of 25 µL/min. This resulted in five individual ligand samples on the GLM-BSA surface with up to approximately 200 RUs of fusion protein being captured for the highest expressing clones. The first ligand channel was left empty for use as a blank control surface. This was immediately followed by two buffer injections to stabilize the baseline in the analyte direction, each for 30 s at a flow rate of 100 µL/min, and then the CEACAM6 analyte injection. Five CEACAM6 concentrations (50, 16.7, 5.6, 1.85, and 0.62 nM) and buffer were simultaneously injected in individual analyte channels at 50 µL/min for 120 s with a 600 s dissociation, resulting in a set of binding sensorgrams with a buffer reference for each of the five captured sdAb-ABTAG fusions. The BSA surfaces with bound sdAbs were regenerated by an 18 s pulse of 0.85% phosphoric acid at a flow rate of 100 µL/min. Sensorgrams were aligned and double-referenced using the buffer blank injection and the resulting sensorgrams were analyzed using ProteOn Manager software v2.1.1. Kinetic parameters were determined by fitting the referenced sensorgrams to a 1:1 interaction model using local *R*_max_, and affinity constants (*K*_D_, nM) were derived from the resulting rate constants [*k*_d_ (s^−1^)/*k*_a_ (M^−1^ s^−1^)].

### SPR Analysis of CEACAM6 Binding to Covalently Immobilized sdAbs and sdAb-ABTAG Fusions

Purified sdAbs identified by conventional panning and the highest affinity binders identified *via* ABTAG screening after one and two rounds of panning were subjected to standard SPR analysis. Following purification by immobilized-metal affinity chromatography (18), sdAbs and sdAb-ABTAG fusions were further purified by size exclusion chromatography (SEC) using Superdex S75 and Superdex S200 Increase columns (GE Healthcare), respectively. CEACAM6 was subjected to Superdex S75 chromatography to remove possible aggregates.

sdAbs from conventional panning and from ABTAG round 2 screening were immobilized on CM5 sensor chips (GE Healthcare) at 50 µg/mL in 10 mM acetate buffer, pH 4. Multiple cycle kinetics were performed on a Biacore 3000 (GE Healthcare) in 10 mM HEPES, pH 7.4, containing 150 mM NaCl, 3 mM EDTA, and 0.005% P20 by flowing appropriate concentrations of CEACAM6 at a flow rate of 40 µL/min. Purified sdAb-ABTAG fusions from ABTAG round 1 screening were immobilized on CM5 sensor chips (GE Healthcare) at concentrations of 12–25 µg/mL in 10 mM acetate buffer, pH 3.5. Single cycle kinetics (SCK) were performed on a Biacore 3000 (GE Healthcare) in 10 mM HEPES, pH 7.4, containing 150 mM NaCl, 3 mM EDTA, and 0.005% P20 by flowing appropriate concentrations of CEACAM6 at a flow rate of 25 µL/min. Data were analyzed using BIAevaluation v4.1.1 Software (GE Healthcare).

### Next-Generation DNA Sequencing (NGS)

The phage-displayed immune llama V_H_H library was interrogated using an Illumina MiSeq instrument as previously described (1, 2). Briefly, replicative form DNA was purified from phagemid-bearing *E. coli* TG1 cells using a QIAprep^®^ spin miniprep kit (Qiagen Inc.). Amplicons for NGS were prepared using PelB- and FR4-specific barcoded primers using ~25 ng of phagemid DNA as template and purified by gel extraction followed by solid-phase reversible immobilization using Agencourt^®^ AMPure^®^ XP beads (Beckman-Coulter, Pasadena, CA, USA). The data were quality filtered using the FAST-X toolkit with a stringency of Q30 over 95% of the read.

## Results

### Selection of CEACAM6-Specific Binders by Conventional Panning

Immunization of a llama with recombinant CEACAM6 resulted in strong immune response against the immunogen as indicated by ELISA comparing the preimmune and postimmunization sera collected on day 71 from the animal (Figure S1 in Supplementary Material).

A phage-displayed V_H_H library with a functional size of 2.9 × 10^7^ clones was constructed from the lymphocytes of the immunized llama and used to select CEACAM6-binding sdAbs. After three rounds of panning, randomly picked clones were tested by phage ELISA to identify clones displaying CEACAM6-specific sdAbs. DNA sequencing revealed that the positive clones were comprised of five different sequences. The unique clones were named 1B6, 1F6, 2A7, 2F8, and 2G9. After subcloning in an expression vector, all five sdAbs were expressed in *E. coli* periplasms and purified. ELISA showed that all five sdAbs bound to CEACAM6 (data not shown). The actual affinities of these sdAbs were determined to be in the 2–10 nM range by SPR (Table 1; Figure S2A in Supplementary Material).

**Table 1:** Kinetic and affinity data for purified sdAbs binding CEACAM6.

sdAb	Source	RUs immobilized	*k*_a_ (M^−1^ s^−1^)	*k*_d_ (s^−1^)	*K*_D_ (nM)
1B6	Conventional panning	1,004	3.48 × 10^5^	8.73 × 10^−4^	2.5
1F6	Conventional panning	257^a^	3.68 × 10^6b^	8.90 × 10^−3b^	2.4^b^
2A7	Conventional panning	900	7.78 × 10^5^	8.34 × 10^−3^	10.7
2F8	Conventional panning	n.d.	n.d.	n.d.	n.d.
2G9	Conventional panning	1,125	7.46 × 10^5^	5.27 × 10^−3^	7.1
2-03	2 rounds + ABTAG	2,221	1.43 × 10^6^	1.17 × 10^−3^	0.8
2-15	2 rounds + ABTAG	1,530	6.01 × 10^5^	3.00 × 10^−4^	0.5
2-35	2 rounds + ABTAG	2,477	1.37 × 10^6^	1.02 × 10^−3^	0.7
1-04	1 round + ABTAG	2,157	2.45 × 10^6^	2.58 × 10^−4^	0.105
1-07	1 round + ABTAG	1,012	3.78 × 10^6^	1.73 × 10^−4^	0.046
1-09	1 round + ABTAG	1,875	1.94 × 10^6^	7.76 × 10^−6c^	0.004
1-19	1 round + ABTAG	1,388	2.69 × 10^6^	6.24 × 10^−4^	0.232

*n.d., not determined*.

*^a^RUs of sdAb-ABTAG captured on BSA surface*.

*^b^Determined using full kinetic ProteOn screen*.

*^c^Approximate *k*_d_ (2 h dissociation of ~4% and at instrument limit of detection)*.

### sdAb-ABTAG Binding to Immobilized BSA

The affinity of ABTAG for BSA, as a fusion with an anti-CEACAM6 sdAb, was determined to be 21 pM with a dissociation rate constant, *k*_d_, of 9.1 ± 0.7 × 10^−6^ s^−1^ (Figure 1D). These values are essentially identical to those previously reported for ABTAG (11). As the dissociation rate is extremely slow, it was determined over a long, 10,000 s, dissociation phase.

### Dissociation Rate and Expression Screening of sdAb-ABTAG Fusion Proteins

**Figure 2:**
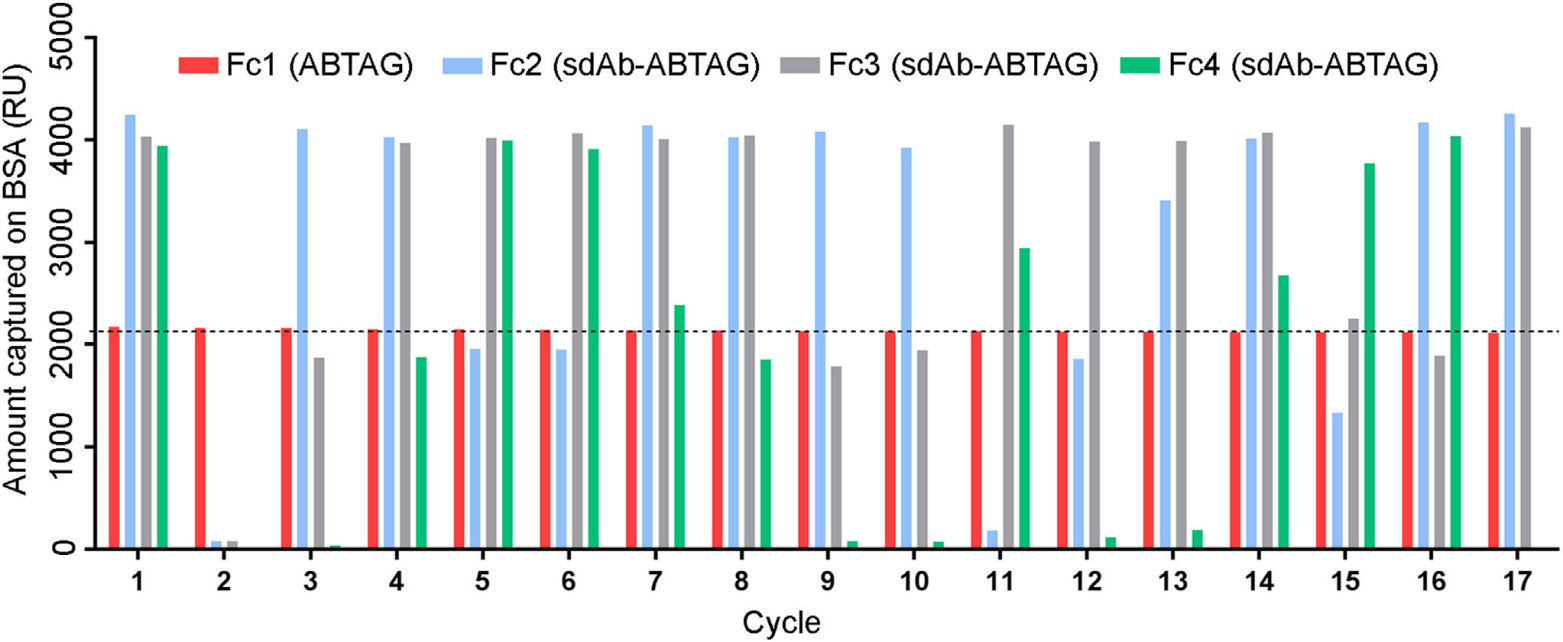
Multiple cycles of ABTAG and single-domain antibody (sdAb)-ABTAG fusion proteins capture on bovine serum albumin (BSA) surfaces for CEACAM6 binding. Capture levels of ABTAG and sdAb-ABTAG fusions over 17 cycles on a Biacore 3000 instrument. The dashed line represents the mean ABTAG capture level on flow cell (Fc) 1. Fc2: flow cell 2; Fc3: flow cell 3; Fc4: flow cell 4.

**Figure 3:**
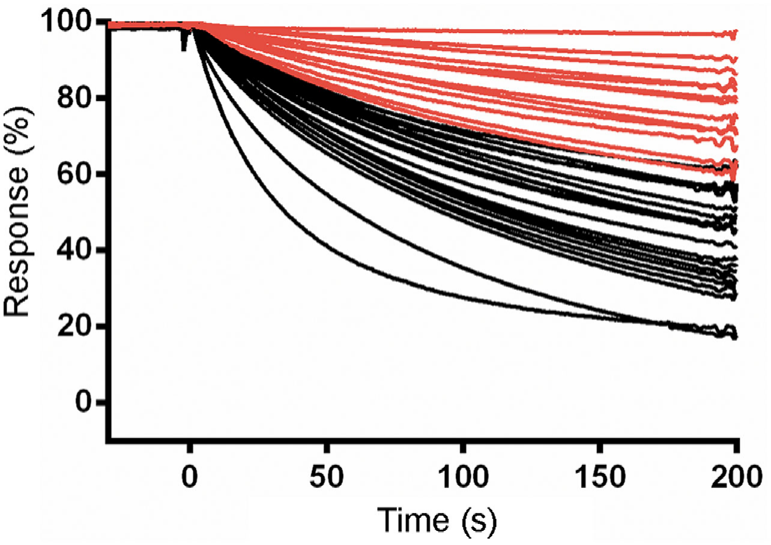
Surface plasmon resonance (SPR) characterization of anti-CEACAM6 single-domain antibodies (sdAbs) obtained by off-rate screening of the round 2 sublibrary. Normalized dissociation phases of SPR sensorgrams of CEACAM6 binding to captured sdAb-ABTAG fusions derived from Biacore 3000 experiments. Red lines represent the sdAb-ABTAG clones with the slowest off-rates.

Eluted phage from the second round of panning were amplified by PCR and cloned into the ABTAG fusion vector (Figures 1B,C). The size of this sublibrary was 1.4 × 10^7^ independent transformants. Individual clones from this sublibrary were grown in microtiter plates and the culture supernatants were used to rank the dissociation rate constants by SPR analysis. A total of 17 cycles were performed to generate the binding data for 48 clones; two duplicates and a buffer injection were included (Table S1 in Supplementary Material). Surface regeneration between cycles was exceedingly efficient with identical amounts of ABTAG being captured on the reference Fc over the 17 cycles (Figure 2). Of the 48 clones, 19 were excluded from off-rate screening and sequence determination: (i) six because the sdAb-ABTAG capture levels were less than 100 RUs, (ii) 11 because there was insignificant antigen binding to the captured fusion proteins and (iii) two because of low sdAb-ABTAG capture levels and poor 1:1 fitting of antigen binding data. As shown in Figure 3 and Table S1 in Supplementary Material the clones exhibited a wide range of off-rates (*k*_d_s), namely, from 1.48 × 10^−4^ to 1.67 × 10^−12^ s^−1^. As the clones were randomly picked, they were not all unique, accounting for the nearly identical dissociation profiles of some of the clones. As the screening measurements were performed at a single CEACAM6 concentration, it was impossible to calculate accurate on-rates and, consequently, accurate *K*_D_ values. Of 29 sequenced clones, one of the clones isolated by conventional panning, 2A7, was the most frequent, accounting for 9 of the 29 clones. Another clone isolated by conventional panning, 2G9, occurred twice. The remaining three sdAbs identified by the conventional approach (1F6, 2F8, and 1B6) were not found by the off-rate screening method. Three clones with the slowest off-rates (2-03 = 6.87 × 10^−4^ s^−1^; 2-15 = 1.48 × 10^−4^ s^−1^; 2-35 = 8.56 × 10^−4^ s^−1^) were subcloned into the pSJF2H expression vector and the sdAbs were purified for full kinetic analysis over a range of antigen concentrations. These three unique sdAbs exhibited affinities below 1 nM (0.5–0.8 nM), up to 10 times stronger than the affinities of the binders selected by conventional panning (Table 1; Figure S2B in Supplementary Material).

The ABTAG/sdAb-ABTAG capture step, prior to antigen flow and dissociation rate ranking, gave expression level data for the 48 clones. The total RUs captured for the various clones were a good measure of relative expression levels (Table S1 and Figure S3 in Supplementary Material). It was possible to quantify the concentrations of sdAb-ABTAG fusions in the culture supernatants. A plot of fusion protein concentration *vs* initial fusion protein binding rate was generated from the linear, mass transport-limited, portions of the sensorgrams (Figure S3 in Supplementary Material). This plot was used to derive fusion protein concentrations in culture supernatants. Approximately half of the clones expressed protein in the 100–600 nM range in microtiter plate cultures (Table S1 in Supplementary Material).

### Full Kinetic and Affinity Screening of sdAb-ABTAG Fusion Proteins

A limitation of the dissociation rate screening with the Biacore 3000 instrument is that single antigen concentrations are utilized for screening and clones must be expressed as pure sdAbs to generate full kinetic information. In view of this, we explored two approaches for obtaining full kinetic data using microtiter plate cultures containing sdAb-ABTAG fusions.

**Figure 4:**
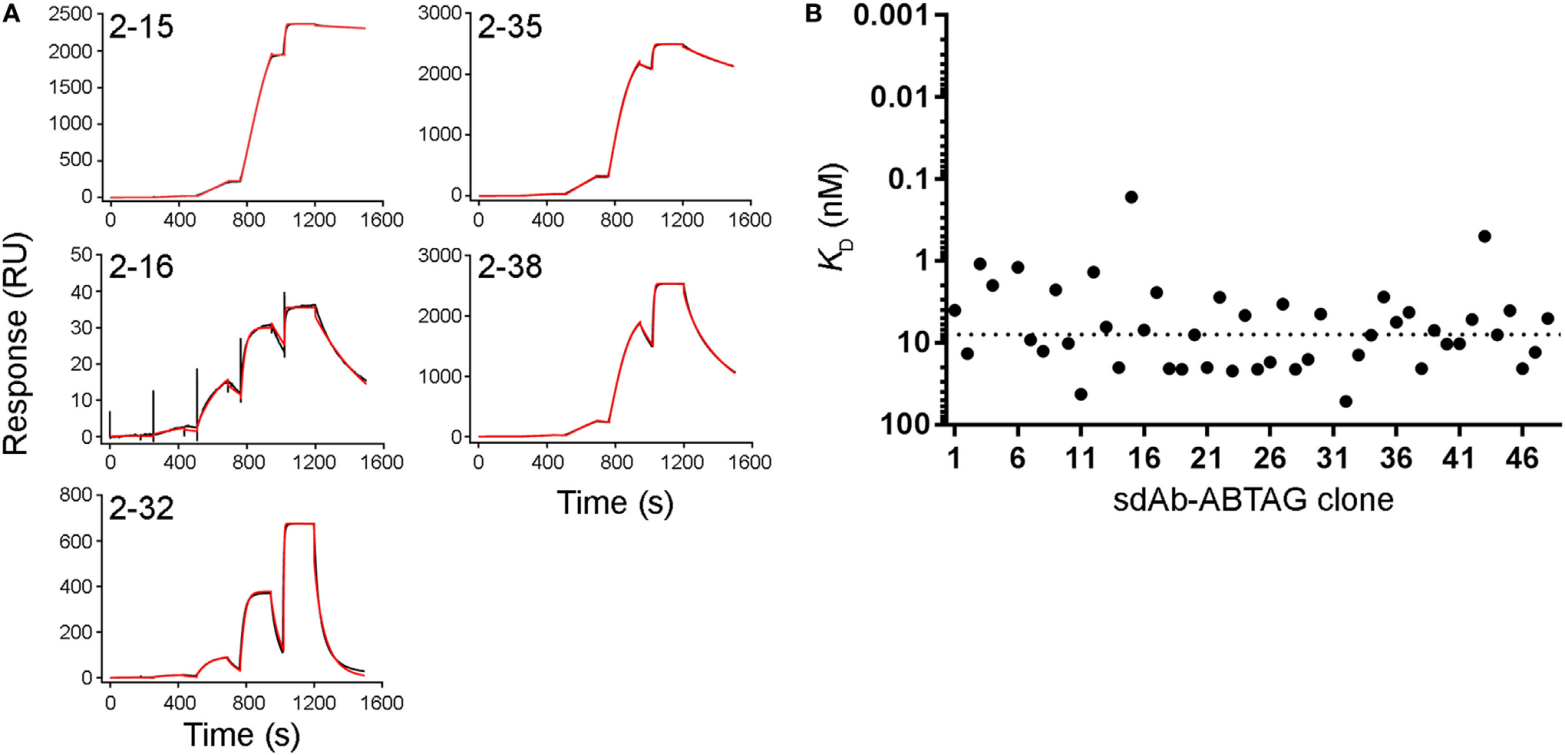
Full kinetic screening of anti-CEACAM6 single-domain antibodies (sdAbs) by single cycle kinetics analysis of the round 2 sdAb-ABTAG sublibrary. Fitting of representative sensorgrams to a 1:1 interaction model **(A)**. Black lines represent raw data; red lines represent 1:1 fits. *K*_D_s derived from Biacore T200 sensorgrams of the binding of five concentrations of CEACAM6 (0.1–1,000 nM) to captured sdAb-ABTAG fusions **(B)**. The dashed line represents the median *K*_D_ = 7.98 nM.

In the first approach SCK experiments were performed in which increasing concentrations of CEACAM6 were flowed over captured sdAb-ABTAG fusions using a Biacore T200 instrument. Microtiter plate periplasmic extracts from the 48 clones from round 2 subjected to off-rate screening were screened by SCK to determine *k*_a_, *k*_d_, and *K*_D_ (Table S2 in Supplementary Material). A wide range of CEACAM6 concentrations (0.1–1,000 nM) was flowed over the captured clones in order to obtain approximate *K*_D_s for clones with wide-ranging affinities as the off-rate screening of the 48 clones gave *k*_d_s covering two orders of magnitude (Table S1 in Supplementary Material). As expected, the concentration range was not ideal for individual clones but in most instances two or three of the concentrations gave acceptable responses (Figure 4A). Fitting the sensorgrams to a 1:1 interaction model gave a surprisingly good approximation of the rate constants and *K*_D_s (Figure 4B; Table S2 in Supplementary Material), as indicated by the rate constants and affinities of some of the clones determined by more rigorous analyses (Table 1). The median *K*_D_ for these analyzed clones was 7.98 nM (Figure 4B).

**Figure 5:**
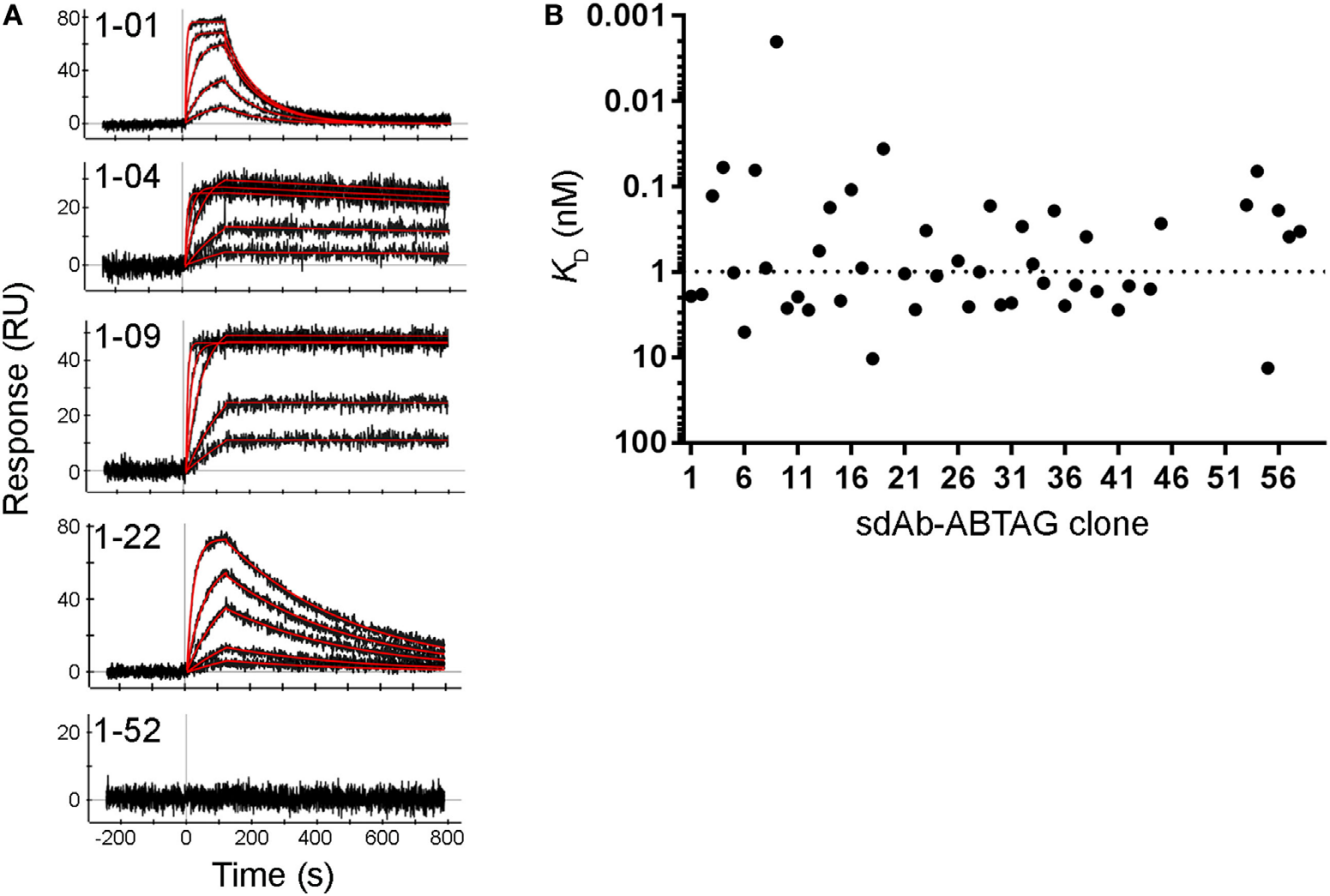
Full kinetic screening of anti-CEACAM6 single-domain antibodies (sdAbs) by multiple cycle kinetics analysis of the round 1 sublibrary. Fitting of representative sensorgrams to a 1:1 interaction model **(A)**. Black lines represent raw data; red lines represent 1:1 fits. *K*_D_s derived from ProteOn sensorgrams of the binding of five concentrations of CEACAM6 (0.62–50 nM) to captured sdAb-ABTAG fusions **(B)**. The dashed line represents the median *K*_D_ = 0.99 nM.

**Figure 6:**
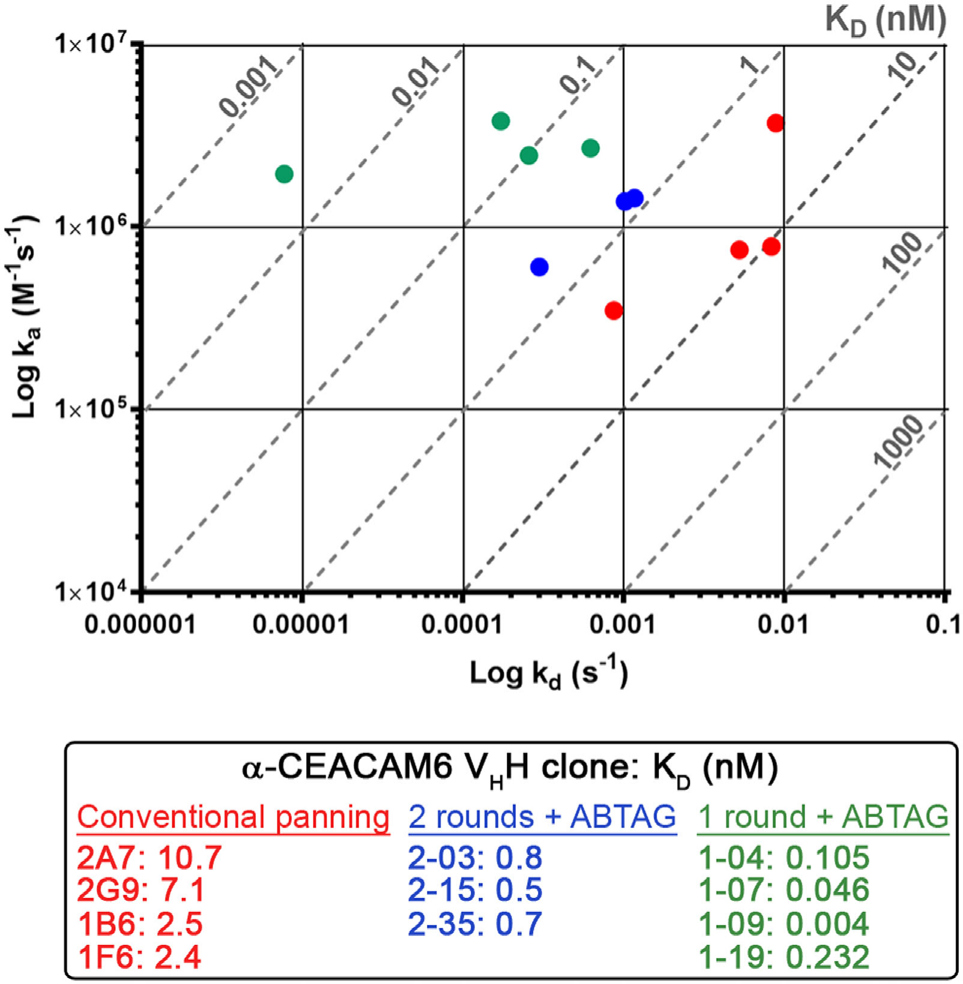
Iso-affinity plots of the highest affinity single-domain antibodies (sdAb) clones obtained using conventional panning or sdAb-ABTAG screening. Reported *K*_D_s were obtained from purified sdAbs or sdAb-ABTAG fusions immobilized on sensor chips and subsequent binding of various concentrations of CEACAM6.

In the second approach, five different CEACAM6 concentrations were flowed over five or six captured sdAb-ABTAG fusions in a single injection cycle using a BioRad ProteOn, which has a 6 by 6 grid of microfluidic channels. To take full advantage of these higher throughput approaches, we made a second, more diverse, sublibrary by cloning phage-displayed sdAbs eluted from round 1 panning into the ABTAG-fusion protein expression vector. Of the 576 randomly picked clones from the round 1 sublibrary, 52 were positive for antigen binding by ELISA (data not shown). Microtiter plate culture supernatants containing sdAb-ABTAG fusions from the 52 positive ELISA clones were screened using a ProteOn to determine sdAb-ABTAG capture levels and, by fitting the sensorgram data to a 1:1 interaction model, rate constants, *K*_D_s and observed R_max_s (Table S3 in Supplementary Material). No antigen binding was observed for several of the clones—low capture levels and low affinities may account for this observation. Three of the five clones identified by conventional panning (2A7, 2G9, and 1F6) were found in this screen. Eleven of the 52 clones were 2G9, three or four were 2A7, and one was 1F6. Of the remaining 36 clones, 32 were unique; four clones were found twice. The sensorgram data were generally of high quality, fitting well to a 1:1 interaction model (Figure 5A). The method gave excellent *K*_D_ approximations (Figure 5B), as evidenced by the values obtained for clones that were subjected to a more stringent SPR analysis, i.e., the sdAbs that were analyzed by binding antigen to immobilized sdAbs (Table 1). The median K_D_ for these analyzed clones was 0.99 nM (Figure 5B). Values are not given for clones with capture levels of less than 100 RUs. Several of the clones had higher affinities, attributable to slower off-rates, than those of clones from the round 2 library. While the very slow ABTAG *k*_d_ results in stable capture of sdAb-ABTAG fusions, direct binding assays in which the ABTAG fusions are covalently linked to the surface and antigen is flowed are preferable in instances where the sdAb *k*_d_ is very slow. While the off-rates determined by the capture method were derived using double referencing (reference cell and zero-concentration subtraction), accurate determination of very slow off-rates can be challenging, even in the best of circumstances. The four round 1 library clones showing the highest affinities by ProteOn screening were expressed and purified as sdAb-ABTAG fusions for direct binding assays (Table 1; Figure S2C in Supplementary Material). Monomeric CEACAM6 peaks from SEC (Figure S2D in Supplementary Material) were used for analysis. The affinities of these four clones, which ranged from 4 to 232 pM (Table 1), were all higher than the affinities of the top round 2 clones (Figure 6; Table S2 in Supplementary Material) with clone 1-09 exhibiting an exceedingly slow off-rate (Table 1). The 1-09 sdAb-ABTAG surface required stringent regeneration conditions (Figure S2C in Supplementary Material).

### Next-Generation DNA Sequencing

To better understand why some of the highest-affinity sdAbs against CEACAM6 were not recovered through conventional panning of the phage-displayed V_H_H library, we interrogated the library to moderate depth (approximately 2.5 × 10^5^ quality-filtered sequences) using amplicon sequencing on an Illumina MiSeq instrument (Table 2). Both the medium-affinity sdAbs recovered *via* conventional panning as well as the extremely high-affinity ones recovered only *via* ABTAG screening were observed in the library at roughly similar frequencies. As panning progressed, the frequencies of all CEACAM6-specific sdAbs increased, indicating that all of the sdAb-phages bound plate-adsorbed CEACAM6 and were eluted from it under high pH. However, several medium-affinity sdAbs (in particular 2A7 and 2G9) rapidly outcompeted the higher-affinity sdAbs, becoming enriched 50–100-fold in the first round of panning and making up the majority of CEACAM6-specific sdAbs by round 2. Thus, utilizing a high-throughput screening technology was necessary in this case to recover the most desirable antibody specificities.

**Table 2:** Frequencies of anti-CEACAM6 sdAbs in the phage-displayed immune V_H_H library and after each of two panning rounds.

sdAb	Source	*K*_D_ (nM)	Library (%)^a^	Round 1 (%)	Round 2 (%)
1B6	Conventional panning	2.5	0.01	n.f.^b^ (2.2)^c^	n.f.^b^
1F6	Conventional panning	2.4	0.07	0.17^b^ (2.2)^c^	n.f.^b^ (4.2)^c^
2A7	Conventional panning	10.7	0.04	0.7^b^ (2.2)^c^	18.9^b^ (41.7)^c^
2F8	Conventional panning	n.d.	0.01	n.f.^b^ (2.2)^c^	n.f.^b^ (4.2)^c^
2G9	Conventional panning	7.1	0.02	1.9^b^	4.2^b^ (29.2)^c^
2-03	2 rounds + ABTAG	0.8	0.01	n.f.^b^	4.2^b^
2-15	2 rounds + ABTAG	0.5	0.005	n.f.^b^	2.1^b^
2-35	2 rounds + ABTAG	0.7	0.01	n.f.^b^	2.1^b^
1-04	1 round + ABTAG	0.105	0.03	0.17^b^	n.f.^b^
1-07	1 round + ABTAG	0.046	0.03	0.17^b^	n.f.^b^
1-09	1 round + ABTAG	0.004	0.005	0.17^b^	n.f.^b^
1-19	1 round + ABTAG	0.232	0.005	0.17^b^	n.f.^b^

*n.d., not determined; n.f., not found*.

*^a^Determined using next-generation DNA sequencing*.

*^b^Determined using Sanger sequencing of ABTAG sublibrary clones*.

*^c^Determined using Sanger sequencing of phage-displayed library clones obtained through conventional panning*.

## Discussion

The present study has shown that ABTAG can be a useful addition to the toolbox for antibody discovery by *in vitro* display methods. Compared to conventional phage display approaches to antibody isolation, ABTAG technology yielded a much higher number of unique sequences while simultaneously giving detailed antigen binding information. With the rapid rise in antibody drugs in recent years, the number of different applications for this class of biologics is ever increasing. Along with each application is also a desirable set of kinetic parameters and functional properties (19). For instance, naked inhibitory mAbs targeting a surface receptor would preferentially have tight-binding with slow off-rates. Conversely, tumor-imaging molecules, or those carrying a toxic payload, require shorter half-lives and faster off-rates. Selection of antibodies based on kinetics using a full kinetics screening method, such as the ProteOn methods described here, is an obvious advantage, and affords a moderate throughput of up to 200 clones per day, extrapolating from the time required to screen the 52 round 1 clones described here, with generation of high quality kinetic and affinity data. Maximizing the number of clones available for functional screening is also clearly a key aspect of the search for antibodies with desired functionalities such as cross-species reactivity and binding to specific epitopes, for example, epitopes that deliver activation, inhibitory, internalization or no biological activity. The ABTAG technology supports the growing trend away from largely blind selection of limited numbers of binders as evidenced, for example, by the use of next generation sequencing to more effectively capture the full diversity of the immune response and bypass B-cell or antibody library screening (1, 20).

Other affinity tags are available for use in purification and in screening technologies such as SPR; however, none of these tags are ideal. The use of streptavidin requires chemical or enzymatic biotinylation of the proteins being screened; SPR screening of biotinylated proteins is not always straightforward and rapid. His-tagged proteins can be captured on Ni^2+^-surfaces but the capture is not always stable enough for the analysis of high affinity interactions; assays require steps for Ni^2+^ activation and regeneration which slows screening throughput and Ni^2+^-chelation surfaces are relatively expensive. His-tagged proteins can be captured by anti-His-tag antibodies but commercial sources of these antibodies can be costly and the antibodies can have variable binding affinity depending on the presentation of the His-tag in the context of the protein. Monoclonal antibodies can be captured on anti-Fc surfaces but the use of Fcs as fusion tags generates homodimeric fusion proteins which are undesirable for some assays. The ABTAG capture system described here is in many ways ideal: (i) the capture molecule (BSA) is readily available, inexpensive and stable on SPR surfaces, (ii) there is no indication that ABTAG negatively impacts the expression or activity of the molecules being screened, (iii) the BSA-ABTAG interaction is of very high affinity due to a very slow off-rate, and (iv) the BSA-ABTAG interaction can be completely reversed without any damage to the BSA surface.

Single-domain antibody-ABTAG screening identified 53 anti-CEACAM6 sdAbs that were not found by a conventional phage display panning approach. The five sdAbs identified by conventional panning grouped into three families—the 2A7/2G9 family, the 1F6/2F8 family, and 1B6. Of the 32 round 2 sublibrary sdAbs, 23 belonged to the 2A7/2G9 family (12 were 2A7 or 2G9) and two belonged to the 1F6/2F8 family. The remaining seven were characterized by longer CDR3s, up to 24 residues; one of these clones was 2-15, which was the round 2 clone with the highest affinity and slowest off-rate (Table 1). Of the 52 round 1 sublibrary sdAbs, 20 belonged to the 2A7/2G9 family (11 were 2G9 and three or four were 2A7—there was some sequence ambiguity) and one belonged to the 1F6/2F8 family. The remaining 31 were characterized by CDR3 lengths of 6–25 residues. The CDR3 lengths of clones 1-04, 1-07, 1-09, and 1-19 were 12, 11, 16, and 12 residues, respectively. Clones 1-04, 1-07, 1-09, and 1-19 had off-rates in the 6.24 × 10^−4^ to 7.76 × 10^−6^ s^−1^ range (Table 1; Figure 2) and required stringent surface regeneration conditions (Figure S2C in Supplementary Material). It is unclear why the group of high-affinity, long-CDR3 sdAbs was less efficiently enriched by panning than the 2A7/2G9 family of medium-affinity, short-CDR3 sdAbs given that no obvious bias in their starting frequencies was observed in the library. We speculate that this observation may relate to the very slow off-rates of the former group of sdAbs which may translate into less efficient elution of these sdAb-phages by high pH; however, a stochastic element to the panning process cannot be ruled out. Clearly, as has been the experience of many groups, phage display selection is ineffective in discriminating antibodies based on affinity and probably depends instead on other factors (e.g., starting frequency; fast off-rates; pH-sensitive binding; growth advantages in *E. coli*). The capture levels (Response; RUs) of the 2A7/2G9 moderate-affinity family and the rarer set of high-affinity clones (1-04, 1-07, 1-09, and 1-19) were very similar (Table S3 in Supplementary Material), suggesting, at least at the protein level, there were no significant differences in the expression of these sdAbs in *E. coli* supernatants. Capture levels (Response; RUs) were clearly shown to correlate with sdAb-ABTAG concentrations (Figure S3 and Table S1 in Supplementary Material). However, it is unknown whether the display efficiency of these sdAbs on the surface of phage is comparable to their soluble expression.

Another benefit of the screening method described here is that both *E. coli* culture supernatants and periplasmic extracts of microtiter plate cultures can be used for ABTAG SPR-based screening. Culture supernatants were used directly for off-rate screening and periplasmic extracts were used for SCK screening of the 48 round 2 clones. Similar capture levels were observed for individual clones with both sdAb-ABTAG sources.

The screening of up to 10^4^ clones in a week by the approach described here is realistic, provided that antigen cost is not prohibitive. This represents very good sampling of the phage clones eluted after a single round of panning, typically 10^5^–10^6^. Of the methods described here, the ProteOn method has the highest throughput, followed by the off-rate screening method and the SCK method, although the throughput potential of the latter two methods is not that different. Analysis of about 30 clones a day is possible with the SCK method. The ProteOn instrument used here has recently been discontinued and will be supported only until 2020. However, as newer higher throughput instruments become available, ABTAG screening throughput equaling or exceeding that achieved here with the ProteOn should not be an issue (21).

Beyond its usefulness in screening sdAb libraries, ABTAG has been applied to other antibody and protein engineering projects (JZ, unpublished data). Both scFv and Fab libraries, including humanization and affinity maturation libraries, can be screened in a manner similar to that described here for sdAbs. In addition to screening for affinity, ABTAG technology can be applied to screening for protein stability, an unmet need in the field of protein engineering. When pure to very pure proteins are needed ABTAG can be used as a purification tag for BSA-affinity matrix purification. When the efficacy of a protein needs to be evaluated *in vivo*, the protein-ABTAG can be mixed with BSA to overcome the limitation of short serum half-life of the protein. Finally, screening libraries constructed from other proteins, receptors, ligands or enzymes, for which binding partners are available, could also be exploited with ABTAG. In summary, ABTAG technology provides a powerful and versatile tool for antibody discovery and protein engineering applications.

## Ethics Statement

This study was carried out in accordance with Animal Use Protocols approved by the National Research Council Canada Animal Care Committee.

## Author Contributions

GH and CRM conceived experiments and planned and wrote the manuscript. GH supervised the Biacore analyses and created the figures. TB constructed the libraries, conducted the panning and coordinated the off-rate and ProteOn screening of the round 1 and round 2 library clones. JB devised and conducted the ProteOn analyses. HvF conducted the off-rate screening and SPR characterization of the purified sdAbs and sdAb-ABTAG fusions. SR conducted the SCK screening of the round 2 clones. KH performed and analyzed NGS experiments. JZ devised the application of ABTAG to the screening of antibody libraries.

## Conflict of Interest Statement

CM and JZ are inventors on US patent 9,327,022 B2. JZ is an inventor on US patent 9,476,887 B2. All other authors declare that the research was conducted in the absence of any commercial or financial relationships that could be construed as a potential conflict of interest.
