# Supplementary material for: A Novel Affinity Tag, ABTAG, and Its Application to the Affinity Screening of Single-Domain Antibodies Selected by Phage Display
Source: Front Immunol. 2017 Oct 30;8:1406. doi: 10.3389/fimmu.2017.01406 (PMC5674936; doi:10.3389/fimmu.2017.01406)
Supplement: Supplementary file 1 [file data_sheet_1.pdf]

*Supplementary Material*

# A Novel Affinity Tag, ABTAG, and its Application to the Affinity Screening of Single-Domain Antibodies Selected by Phage Display

Greg Hussack[1*], Toya Nath Baral[1,a], Jason Baardsnes[2], Henk van Faassen[1], Shalini Raphael[1], Kevin A Henry[1], Jianbing Zhang[1,b*] and C. Roger MacKenzie[1]

[1]Human Health Therapeutics Research Centre, National Research Council Canada, 100 Sussex Drive, Ottawa, Ontario, Canada K1A 0R6,
[2]Human Health Therapeutics Research Centre, National Research Council Canada, 6100 Royalmount Avenue, Montréal, Québec, Canada H4P 2R2

*Correspondence:
Greg Hussack
Greg.Hussack@nrc-cnrc.gc.ca
Jianbing Zhang
jianbing.oliver@outlook.com

[a]Present Address: Merck and Company Inc., Palo Alto, CA, USA
[b]Present Address: Xiangxue Pharma, Jinfengyuan Road 2, Huangpu District, Guangzhou, China 510663

## Supplementary Tables

**Supplementary Table 1.** Summary of data obtained by off-rate screening of randomly picked sdAb-ABTAG clones from the round 2 sub-library.

| Cycle | sdAb | Conventional Panning Designation | sdAb-ABTAG Captured (RUs) | $k_d$ ($s^{-1}$) | sdAb-ABTAG Concentration (nM)[a] |
|---|---|---|---|---|---|
| 1 | 2-01 | | 4245 | 1.14E-03 | 261 |
| 17 | 2-01 | | 4258 | 1.15E-03 | 254 |
| 1 | 2-02 | | 4030 | 4.09E-03 | 609 |
| 1 | 2-03[b] | | 3944 | 6.87E-04 | 485 |
| 2 | 2-04 | | 81 | - | 7 |
| 2 | 2-05[b] | | 79 | - | 15 |
| 2 | 2-06 | | 8 | - | 8 |
| 3 | 2-07 | | 4103 | 3.01E-03 | 229 |
| 3 | 2-08 | | 1868 | - | 47 |
| 3 | 2-09 | | 39 | - | 9 |
| 4 | 2-10 | 2G9 | 4028 | 3.28E-03 | 231 |
| 4 | 2-11 | | 3974 | 7.55E-03 | 492 |
| 4 | 2-12 | | 1873 | - | 43 |
| 5 | 2-13 | | 1952 | - | 36 |
| 5 | 2-14 | 2A7 | 4022 | 5.17E-03 | 415 |
| 5 | 2-15 | | 3993 | 1.48E-04 | 186 |
| 6 | 2-16 | | 1949 | - | 37 |
| 6 | 2-17 | | 4064 | 4.58E-04 | 149 |
| 6 | 2-18 | 2A7 | 3910 | 5.09E-03 | 446 |
| 7 | 2-19 | 2A7 | 4141 | 5.08E-03 | 334 |
| 7 | 2-20 | | 4006 | 3.34E-03 | 349 |
| 7 | 2-21 | | 2382 | 6.00E-03 | 51 |
| 8 | 2-22 | | 4023 | 7.83E-04 | 335 |
| 8 | 2-23 | 2A7 | 4043 | 5.01E-03 | 479 |
| 8 | 2-24 | | 1853 | - | 43 |
| 9 | 2-25 | 2A7 | 4080 | 4.84E-03 | 361 |
| 9 | 2-26 | | 1785 | - | 40 |
| 9 | 2-27 | | 76 | - | 3 |
| 10 | 2-28 | | 3923 | 6.46E-03 | 110 |
| 10 | 2-29 | | 1943 | - | 47 |
| 10 | 2-30 | | 72 | - | 9 |
| 11 | 2-31 | | 179 | - | 14 |
| 11 | 2-32 | | 4148 | 1.67E-02 | 175 |
| 11 | 2-33 | 2A7 | 2941 | 5.24E-03 | 59 |
| 12 | 2-34 | | 1857 | - | 35 |
| 12 | 2-35 | | 3983 | 8.56E-04 | 517 |
| 12 | 2-36 | | 112 | 9.23E-03 | 4 |
| 13 | 2-37 | | 3407 | 4.77E-04 | 167 |
| 13 | 2-38 | 2A7 | 3992 | 5.19E-03 | 428 |
| 13 | 2-39 | | 190 | - | 6 |
| 14 | 2-40 | | 4016 | 2.81E-03 | 430 |
| 14 | 2-41 | 2G9 | 4071 | 3.22E-03 | 401 |
| 14 | 2-42 | | 2675 | - | 53 |
| 15 | 2-43 | | 1332 | 2.53E-04 | 28 |
| 15 | 2-44 | 2A7 | 2253 | - | 63 |
| 15 | 2-45 | | 3773 | 1.78E-03 | 159 |
| 16 | 2-46 | 2A7 | 4172 | 4.83E-03 | 452 |
| 16 | 2-47 | | 1889 | - | 45 |
| 16 | 2-48 | | 4037 | 1.78E-03 | 290 |
| 17 | 2-48 | | 4120 | 1.85E-03 | 260 |
| 17 | Blank | | 6 | - | - |

[a]See Supplementary Figure 3

[b]2-3 and 2-5 have the same sequence

**Supplementary Table 2.** Summary of data obtained by SCK screening of randomly picked sdAb-ABTAG clones from the round 2 sub-library.

| Cycle | sdAb | Conventional Panning Designation | sdAb-ABTAG Captured (RUs) | $k_a$ ($M^{-1}s^{-1}$) | $k_d$ ($s^{-1}$) | $K_D$ (nM) |
|---|---|---|---|---|---|---|
| 2 | 2-01 | | 3734 | 1.71E+05 | 6.81E-04 | 3.9 |
| 2 | 2-02 | | 4034 | 2.67E+05 | 3.60E-03 | 13.5 |
| 2 | 2-03[a] | | 3555 | 5.64E+05 | 6.10E-04 | 1.1 |
| 4 | 2-04 | | 383 | 3.74E+05 | 7.39E-04 | 2.0 |
| 4 | 2-05[a] | | 13 | - | - | - |
| 4 | 2-06 | | 144 | 4.81E+05 | 5.74E-04 | 1.2 |
| 6 | 2-07 | | 3489 | 2.78E+05 | 2.57E-03 | 9.2 |
| 6 | 2-08 | | 2289 | 3.90E+05 | 4.96E-03 | 12.7 |
| 6 | 2-09 | | 230 | 4.19E+05 | 9.39E-04 | 2.2 |
| 8 | 2-10 | 2G9 | 3383 | 2.90E+05 | 2.94E-03 | 10.1 |
| 8 | 2-11 | | 3885 | 2.14E+05 | 9.03E-03 | 42.2 |
| 8 | 2-12 | | 1990 | 3.86E+05 | 5.28E-04 | 1.4 |
| 10 | 2-13 | | 1983 | 4.25E+05 | 2.72E-03 | 6.4 |
| 10 | 2-14 | 2A7 | 3960 | 2.18E+05 | 4.38E-03 | 20.1 |
| 10 | 2-15 | | 3522 | 3.13E+05 | 5.17E-05 | 0.17 |
| 12 | 2-16 | | 1974 | 3.96E+05 | 2.77E-03 | 7.0 |
| 12 | 2-17 | | 3825 | 1.07E+05 | 2.59E-04 | 2.4 |
| 12 | 2-18 | 2A7 | 3424 | 3.61E+05 | 7.44E-03 | 20.6 |
| 14 | 2-19 | 2A7 | 3379 | 2.89E+05 | 6.10E-03 | 21.1 |
| 14 | 2-20 | | 2570 | 3.75E+05 | 3.00E-03 | 8.0 |
| 14 | 2-21 | | 3180 | 4.51E+05 | 9.07E-03 | 20.1 |
| 16 | 2-22 | | 3329 | 2.02E+05 | 5.62E-04 | 2.8 |
| 16 | 2-23 | 2A7 | 3855 | 2.91E+05 | 6.44E-03 | 22.1 |
| 16 | 2-24 | | 1927 | 2.94E+05 | 1.36E-03 | 4.6 |
| 18 | 2-25 | 2A7 | 3388 | 2.84E+05 | 6.01E-03 | 21.1 |
| 18 | 2-26 | | 2184 | 2.43E+05 | 4.17E-03 | 17.2 |
| 18 | 2-27 | | 338 | 4.43E+05 | 1.50E-03 | 3.4 |
| 20 | 2-28 | | 2553 | 5.17E+05 | 1.09E-02 | 21.0 |
| 20 | 2-29 | | 2176 | 2.88E+05 | 4.59E-03 | 16.0 |
| 20 | 2-30 | | 139 | 3.95E+05 | 1.76E-03 | 4.5 |
| 22 | 2-31 | | 8 | - | - | - |
| 22 | 2-32 | | 2578 | 3.87E+05 | 2.02E-02 | 52.0 |
| 22 | 2-33 | 2A7 | 2272 | 5.38E+05 | 7.61E-03 | 14.1 |
| 24 | 2-34 | | 1910 | 4.04E+05 | 3.25E-03 | 8.1 |
| 24 | 2-35 | | 3701 | 1.95E+05 | 5.35E-04 | 2.8 |
| 24 | 2-36 | | 116 | 1.29E+06 | 7.20E-03 | 5.6 |
| 26 | 2-37 | | 2482 | 8.48E+04 | 3.60E-04 | 4.2 |
| 26 | 2-38 | 2A7 | 3802 | 2.67E+05 | 5.50E-03 | 20.6 |
| 26 | 2-39 | | 1481 | 3.44E+05 | 2.43E-03 | 7.1 |
| 28 | 2-40 | | 3285 | 2.69E+05 | 2.77E-03 | 10.3 |
| 28 | 2-41 | 2G9 | 3691 | 3.58E+05 | 3.64E-03 | 10.2 |
| 28 | 2-42 | | 3390 | 3.25E+05 | 1.68E-03 | 5.2 |
| 30 | 2-43 | | 3164 | 2.36E+05 | 1.17E-04 | 0.50 |
| 30 | 2-44 | 2A7 | 722 | 3.54E+05 | 2.82E-03 | 7.8 |
| 30 | 2-45 | | 3267 | 3.87E+05 | 1.56E-03 | 4.0 |
| 32 | 2-46 | 2A7 | 3326 | 3.07E+05 | 6.31E-03 | 20.6 |
| 32 | 2-47 | | 2147 | 2.77E+05 | 3.59E-03 | 13.0 |
| 32 | 2-48 | | 3370 | 3.22E+05 | 1.62E-03 | 5.1 |

[a]2-3 and 2-5 have the same sequence

**Supplementary Table 3.** Summary of data obtained by full kinetics screening of ELISA-positive sdAb-ABTAG clones from the round 1 sub-library.

| sdAb | Conventional Panning Designation | sdAb-ABTAG Captured (RUs) | $k_a$ ($M^{-1}s^{-1}$) | $k_d$ ($s^{-1}$) | $K_D$ (nM) | Observed $R_{max}$ (RUs) |
|---|---|---|---|---|---|---|
| 1-1 | 2A7 | 227 | 5.1E+06 | 9.7E-03 | 1.9 | 71 |
| 1-2 | | 85 | 6.3E+06 | 1.0E-02 | 1.8 | 17 |
| 1-3 | | 99 | 5.6E+06 | 7.2E-04 | 1.3 | 29 |
| 1-4 | | 88 | 3.5E+06 | 2.1E-04 | 0.06 | 25 |
| 1-5 | 2G9 | 116 | 6.0E+06 | 6.0E-03 | 1 | 33 |
| 1-6 | 2G9 | 95 | 1.6E+05 | 8.2E-04 | 5.1 | 4 |
| 1-7 | | 104 | 3.1E+06 | 2.0E-04 | 0.06 | 29 |
| 1-8 | 2G9 | 82 | 6.9E+06 | 6.2E-03 | 0.9 | 22 |
| 1-9 | | 147 | 3.7E+06 | 6.9E-06 | 0.002 | 47 |
| 1-10[a] | | 78 | 1.8E+06 | 4.8E-03 | 2.6 | 17 |
| 1-11[a] | | 98 | 2.3E+06 | 4.6E-03 | 1.9 | 23 |
| 1-12 | 2G9 | 82 | 6.0E+06 | 2.0E-02 | 2.8 | 19 |
| 1-13[b] | | 31 | - | - | - | 4,NB |
| 1-14 | | 62 | 3.0E+06 | 5.3E-04 | 0.18 | 17 |
| 1-15 | Probably 2A7 | 113 | 6.1E+06 | 1.0E-02 | 2.2 | 33 |
| 1-16[b] | | 88 | 2.7E+06 | 2.9E-04 | 0.1 | 23 |
| 1-17 | | 108 | 8.1E+06 | 7.2E-03 | 0.9 | 30 |
| 1-18 | 2A7 | 129 | 4.5E+06 | 5.0E-02 | 10 | 34 |
| 1-19 | | 311 | 4.3E+06 | 1.6E-04 | 0.04 | 48 |
| 1-20 | 2G9 | -4 | - | - | - | NB |
| 1-21 | 2G9 | 138 | 6.2E+06 | 6.5E-03 | 1 | 40 |
| 1-22 | 2G9 | 242 | 9.2E+05 | 2.5E-03 | 2.8 | 76 |
| 1-23 | | 192 | 1.4E+06 | 4.7E-04 | 0.33 | 16 |
| 1-24 | 2G9 | 241 | 4.2E+06 | 4.7E-03 | 1.1 | 71 |
| 1-25 | | 71 | - | - | - | 3,NB |
| 1-26 | | 73 | 3.3E+06 | 2.4E-03 | 0.7 | 16 |
| 1-27 | | 189 | 9.7E+05 | 2.5E-03 | 2.6 | 59 |
| 1-28 | 2G9 | 237 | 4.4E+06 | 4.3E-03 | 1 | 71 |
| 1-29[c] | | 213 | 1.4E+06 | 2.3E-04 | 0.17 | 13 |
| 1-30 | 1F6 | 257 | 3.7E+06 | 8.9E-03 | 2.4 | 75 |
| 1-31 | | 185 | 5.2E+06 | 1.0E-02 | 2.3 | 58 |
| 1-32 | | 202 | 1.4E+06 | 4.0E-04 | 0.29 | 60 |
| 1-33 | | 206 | 4.4E+06 | 3.6E-03 | 0.8 | 8 |
| 1-34 | | 211 | 2.7E+06 | 3.6E-03 | 1.3 | 57 |
| 1-35 | | 198 | 4.4E+06 | 8.3E-04 | 0.19 | 35 |
| 1-36 | 2A7 | 274 | 4.4E+06 | 1.0E-02 | 2.5 | 85 |
| 1-37 | | 94 | 3.1E+06 | 4.4E-03 | 1.4 | 20 |
| 1-38[d] | | 261 | 4.2E+06 | 1.6E-03 | 0.39 | 84 |
| 1-39[d] | | 257 | 2.6E+06 | 4.4E-03 | 1.7 | 70 |
| 1-40 | | 206 | - | - | - | 1,NB |
| 1-41 | 2G9 | 291 | 3.5E+06 | 9.6E-03 | 2.8 | 86 |
| 1-42 | 2G9 | 304 | 3.3E+06 | 4.8E-03 | 1.5 | 82 |
| 1-43 | | 23 | - | - | - | NB |
| 1-44 | | 177 | 1.7E+06 | 2.7E-03 | 1.6 | 54 |
| 1-45 | | 188 | 4.1E+06 | 1.1E-03 | 0.3 | 58 |
| 1-46[c] | | 250 | - | - | - | 8,NB |
| 1-47 | | 38 | - | - | - | 4,NB |
| 1-48 | | 54 | - | - | - | 4,NB |
| 1-49 | | 29 | - | - | - | 4,NB |
| 1-50 | | 94 | - | - | - | NB |
| 1-51 | | 281 | - | - | - | 5,NB |
| 1-52 | | 25 | - | - | - | NB |

[a]1-10 and 1-11 have the same sequence
[b]1-13 and 1-16 have the same sequence
[c]1-29 and 1-46 probably have the same sequence
[d]1-38 and 1-39 have the same sequence
NB: no binding observed

**Supplementary Figures**

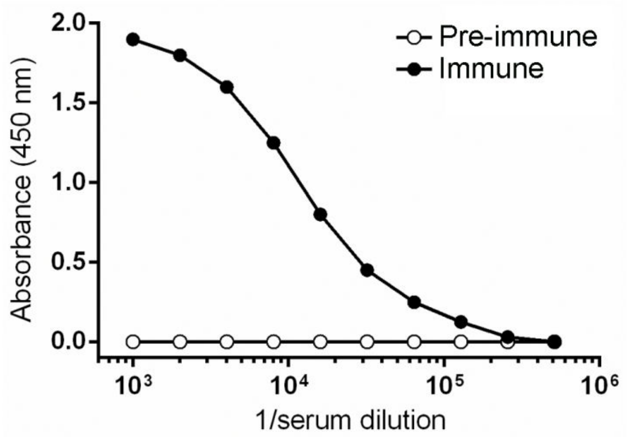


**Supplementary Figure 1.** ELISA comparing pre-immunization and post-immunization (day 71) sera responses from a llama immunized with CEACAM6 extracellular domain (residues 35-232).

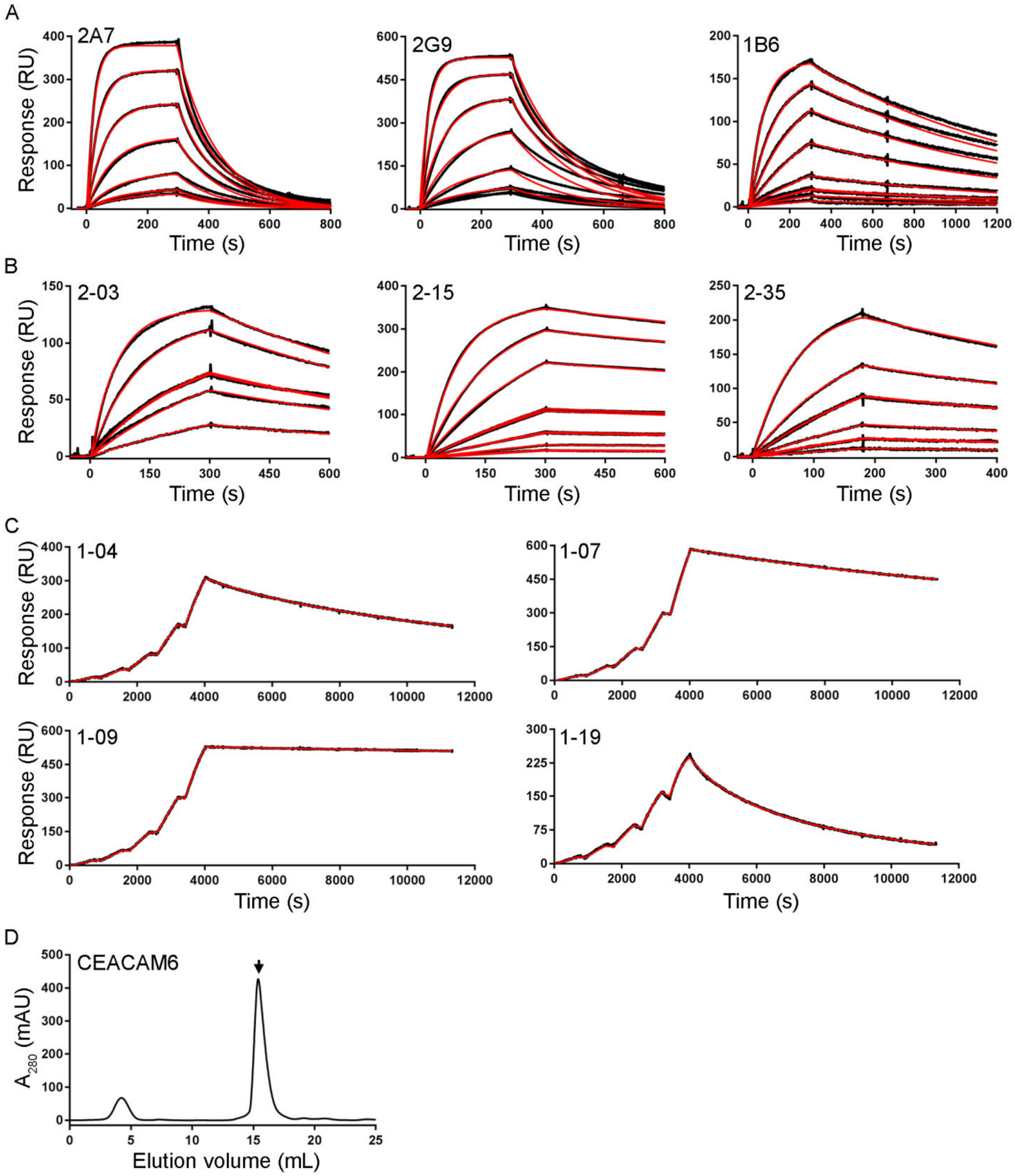


**Supplementary Figure 2.** Sensorgrams showing the binding of CEACAM6 to amine coupled sdAbs and sdAb-ABTAG fusions and fitting of the sensorgram data to a 1:1 interaction model. Black lines represent raw data and red lines represent fitted data. For 2A7 and 2G9, CEACAM6 was flowed at concentrations of 0.25, 5, 1 (duplicates), 2, 5, 10, 20 and 40 nM; for 1B6 at concentrations of 0.75, 1 (duplicates), 2, 5, 10, 20 and 40 nM (A). For 2-03, CEACAM6 was

flowed at concentrations of 0.5, 1, 2 (duplicates), 5, 10 and 20 nM; for 2-15 at concentrations of 0.25, 0.5, 1 (duplicates), 2, 5 and 10 nM and the surface was regenerated with a 6 s pulse of glycine, pH 2.5; for 2-35 at concentrations of 0.25, 0.5, 1, 2 (duplicates), 4 and 10 nM and the surface was regenerated with a 6 s pulse of glycine, pH 2.5 (B). For 1-04, 1-07, 1-09 and 1-19, CEACAM6 was flowed at concentrations of 0.125, 0.25, 0.5, 1 and 2 nM; the 1-04, 1-07 and 1-19 surfaces were regenerated with a 6 s pulse of glycine/HCl, pH 1.5; the 1-09 surface was regenerated with 2 x 4 min pulses of glycine/HCl, pH 1.5 (C). SEC profile of CEACAM6 used for SPR analyses (D).

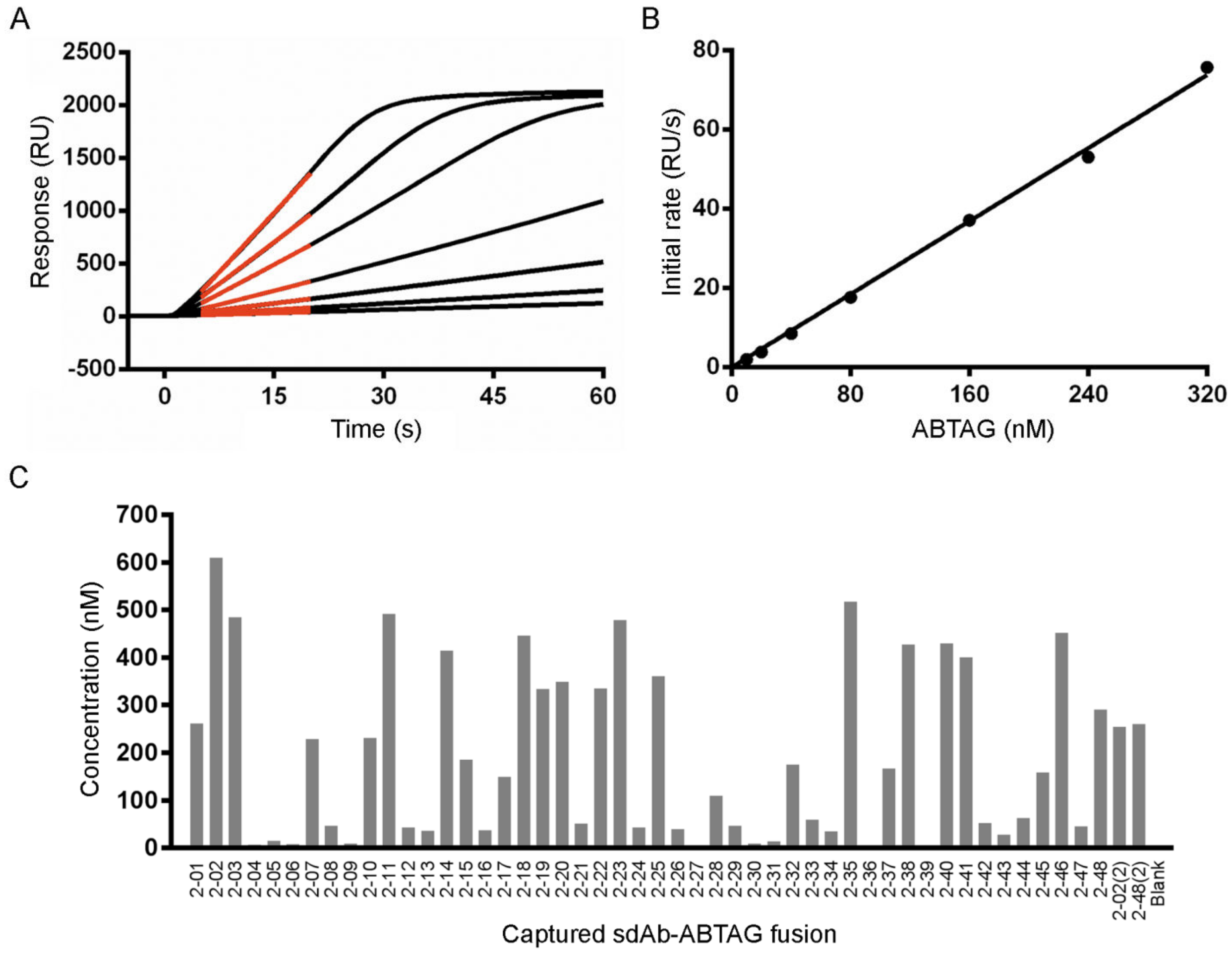


**Supplementary Figure 3.** Quantitation of sdAb-ABTAG fusion proteins in culture supernatants. 10 – 320 nM sdAb-ABTAG binding to immobilized BSA showing the portions of the sensorgrams for standard curve generation in red (A). Standard curve for sdAb-ABTAG fusion protein concentration (B). Total responses for captured sdAb-ABTAG fusions and concentrations derived from initial binding rates (C).
